# Endogenous Propionibacterium acnes Promotes Ovarian Cancer Progression via Regulating Hedgehog Signalling Pathway

**DOI:** 10.3390/cancers14215178

**Published:** 2022-10-22

**Authors:** Qifa Huang, Xin Wei, Wenyu Li, Yanbing Ma, Guanxiang Chen, Lu Zhao, Ying Jiang, Siqi Xie, Qi Chen, Tingtao Chen

**Affiliations:** 1 Department of Obstetrics & Gynecology, The Second Affiliated Hospital of Nanchang University, Nanchang 330006, China; 2 National Engineering Research Center for Bioengineering Drugs and the Technologies, Institute of Translational Medicine, Nanchang University, Nanchang 330031, China

**Keywords:** epithelial ovarian cancer, tumour microbiome, hedgehog signalling pathway, high-throughput sequencing, chronic inflammation

## Abstract

**Simple Summary:**

Epithelial ovarian cancer (EOC) is the most lethal gynaecological malignancy, yet with a still unsatisfactory clinical outcome. Currently, the relationship between intratumor microbiota and the treatment of malignant tumours is a research hotspot, and studies have elucidated their functions on tumour biology. However, there are few studies on the microbial composition of the epithelial benign ovarian tumour or EOC and the potential role of intratumour microbiota in EOC, and its molecular mechanism is still lacking. This study compares the similarities and differences of intratumour microbiota among patients with epithelial benign ovarian tumour and patients with EOC, identifies Propionibacterium acnes is a key strain in facilitating EOC progression, and further ascertains the underlying molecular mechanisms associated with the increased inflammatory response to activate the hedgehog signalling pathway. This could provide insights on improving EOC therapeutics.

**Abstract:**

Background: The oncogenesis and progression of epithelial ovarian cancer (EOC) is a complicated process involving several key molecules and factors, yet whether microbiota are present in EOC, and their role in the development of EOC, remains greatly unknown. Methods: In this study, 20 patients were enrolled to compare the similarities and differences of intratumour microbiota among patients with epithelial benign ovarian tumours (EBOTs) and patients with EOC based on the high-throughput sequencing method. Subsequently, we further isolated the specific EOC-related bacteria and defined *Propionibacterium acnes* as a key strain in facilitating EOC progression. More importantly, we constructed a mouse EOC model to evaluate the effect of the *P. acnes* strain on EOC using immunohistochemistry, Western blotting, and RT-qPCR. Results: The high-throughput sequencing showed that the intratumour microbiota in EOC tissues had a higher microbial diversity and richness compared to EBOT tissues. The abundance of previously considered pathogens, *Actinomycetales*, *Acinetobacter*, *Streptococcus*, *Ochrobacterium*, and *Pseudomonadaceae Pseudomonas*, was increased in the EOC tissues. Meanwhile, we discovered the facilitating role of the *P. acnes* strain in the progression of EOC, which may be partially associated with the increased inflammatory response to activate the hedgehog (Hh) signalling pathway. This microbial-induced EOC progression mechanism is further confirmed using the inhibitor GANT61. Conclusions: This study profiled the intratumour microbiota of EBOT and EOC tissues and demonstrated that the diversity and composition of the intratumour microbiota were significantly different. Furthermore, through in vivo and in vitro experiments, we confirmed the molecular mechanism of intratumour microbiota promotion of EOC progression in mice, which induces inflammation to activate the Hh signalling pathway. This could provide us clues for improving EOC treatment.

## 1. Introduction

Ovarian cancer is regarded as the most lethal gynaecological malignancy, which mainly originates from fallopian tube cancer and peritoneal malignancy [1,2]. Among all subtypes, epithelial ovarian cancer (EOC) is the most prevalent, accounting for over 150,000 deaths each year worldwide [3,4]. In the United States alone, approximately 22,000 new cases are reported annually, posing a great burden for the entire health system [5]. Currently, the standard treatment of EOC includes cytoreductive surgery in combination with platinum-based chemotherapy, radiotherapy, and immunotherapy, from which ovarian cancer patients have greatly benefited. Nevertheless, no treatments lead to a complete cure, and the overall five-year survival rate remains poor (around 30–40%) [6,7]. The high mortality may be attributed to multiple factors, including the underlying aetiology of ovarian cancer, high postoperative recurrence, and existing chemotherapeutic resistance. Therefore, deeper research into the molecular pathogenesis of EOC to search for new therapeutic strategies is urgently needed.

The occurrence and progression of EOC is a complex and multifactorial process involving gene mutations, reconstruction of the tumour microenvironment (TME), dysregulation of multiple signalling pathways, and autophagy [8,9,10]. Among them, the hedgehog (Hh) signalling pathway has an active part in ovarian carcinogenesis and pathogenesis. The Hh signalling pathway promotes the secretion of TGF1 β, binding to TGF-β receptors for the phosphorylation of R-Smad protein, which forms a complex with Smad 4 to enter the nucleus and upregulate Snail 2 expression, ultimately contributing to the development of ovarian cancer [11]. Moreover, Gli1, a nuclear transcription factor of the Hh signalling pathway, can directly induce Snail upregulation and decrease the expression of E-cadherin that facilitates epithelial–mesenchymal transition (EMT) in ovarian cancer, thereby promoting tumour metastasis [12]. Our previous study also indicated the promoting role of Hh in invasion and metastasis via increasing the adhesion molecule CD24 expression [13]. In addition, the Hh signalling pathway also regulates the transcription and translation of MMP-7 expression by the binding of transcription factors Gli1/Gli2 to MMP-7 to drive EOC, revealing the potential significance of Hh signalling in EOC [14].

Accumulating evidence has shown that microbiota is viewed as an increasingly important factor in the initiation and progression of many diseases, including cancer [15,16,17,18]. For the past few years, there has been growing interest in the interaction between microbiota and several malignancies. For example, previous studies have illustrated that significant microbial compositional differences existed between liver cancer patients and patients with cirrhosis, with the number of butyric acid-producing bacteria decreased, while the lipopolysaccharide-producing strains increased significantly, indicating that specific microbiota species may facilitate carcinogenesis [19]. Moreover, with the advent of next-generation sequencing technology, the previous recognition of the sterile TME was challenged and the existence of tumour microbiota was proved and elucidated as functionally importantly in tumourigenesis, including, but not restricted to, pancreatic, breast, and lung cancers [20,21,22,23]. For instance, Straussman et al. using a pancreatic cancer mouse model, showed that intratumoural Gammaproteobacteria could metabolise the chemotherapeutic drug gemcitabine into inactive form, which lead to resistance. However, this phenomenon could be reversed by the use of antibiotics, suggesting that the intratumour microbiota could intercept the anticancer treatment and promote tumourigenesis [24]. Additionally, a study using 16s rRNA gene sequencing techniques on 180 patients with breast cancer reported that a lower bacterial diversity and richness were observed in the TME compared to nontumourous tissue, where the intratumour microbiota were demonstrated to regulate the cytoskeleton and cellular activity under mechanical stress, thereby promoting breast cancer metastasis [25]. Nevertheless, whether the microbiota is present in ovarian tumours, and its role in the development of EOC, remains unclear and needs to be further explored.

In this study, 20 patients were enrolled to characterise the intratumour microbiota among patients with EBOT (Average age: 39.00, *n* = 10) and patients with EOC (Average age: 50.80, *n* = 10) based on the 16s rRNA gene sequencing method. Moreover, bacterial culture and identification were conducted to isolate the specific EOC-related bacteria, which was then intratumourally injected into ID8 cells induced mouse EOC models. The protumourigenic effects of this strain in mice were verified using immunohistochemistry, Western blotting, and RT-qPCR to further ascertain the underlying molecular mechanisms, which could be viewed as strong implications for future EOC treatment.

## 2. Materials and Methods

### 2.1. Enrollment of Patients with EOC and Collection of Tumour Samples

Tumour tissue samples and clinical data were collected from 20 patients with epithelial ovarian tumours admitted from November 2020 to December 2021 (Figure S1). Inclusion criteria were as follows: (1) The patients were aged 18–75 years old and all patients with epithelial ovarian tumours (serous tumour, mucinous tumour) were indicated by imaging and confirmed by postoperative pathology; (2) All patients were in good mental condition, with no change in weight and normal vital signs such as heart rate, breathing, and blood pressure, did not use antibiotics within the past three months, and denied severe heart, lung, kidney, or liver dysfunction and metabolic diseases; (3) Patients with severe comorbidity and previous radiotherapy or chemotherapy treatment were excluded. General anaesthesia was performed for all patients before operation, most of whom received radical resection of epithelial ovarian tumours. Fresh tissues of EBOT and EOC were collected in the sterile surgery room from the Second Affiliated Hospital of Nanchang University and were immediately transferred to sterile 15 mL conical tubes with sterile DMEM culture medium. Samples were processed in the clean and sterile cell culture hood with autoclaved dissection tools, as much as possible, to reduce contamination. Characteristics of patients with epithelial ovarian tumour is shown in Supplementary Table S1.

Patients were divided into EBOT group (ovarian pathological biopsy suggests serous cystadenoma or myxoid cystadenoma, *n* = 10) and EOC group (ovarian pathological biopsy suggests serous cystadenocarcinoma or myxoid cystadenocarcinoma, *n* = 10). All patients in this clinical study were informed in advance, with consent signed before the tumour tissue collection. This study was approved by the ethics committee of the Second Affiliated Hospital of Nanchang University (IRB-2020-061) and registered by the China Clinical Trial Registry (no. ChiCTR2000041320).

### 2.2. DNA Extraction and Bacterial 16S rDNA Sequencing

TIANamp Bacteria DNA Kit (TianGen) was used to extract bacterial genomic DNA based on the manufacturer’s instructions. Concentration and quality of extracted DNA were determined by NanoDrop spectrophotometer. After that, the 16 s ribosomal DNA (rDNA) V4 region was amplified using primers 515F (5′-AYTGGGYDTAAAGNG-3′) and 806R (5′-TACNVGGGTATCTAATCC-3′), and the PCR products were sequenced on the IlluminaHiSeq 2000 platform (Illumina, Inc., San Diego, CA, USA). Paired end reads from the original DNA fragments were processed using Cut Adapt (version 1.9.1. Available online: http://cutadapt.readthedocs.io/en/stable/, accessed on 19 April 2022) and UCHIME Algorithm (Available online: http://www.drive5.com/usearch/manual/uchime_algo.html, accessed on 19 April 2022). Sequencing analysis was subsequently performed by UPARSE software package (version 7.0.100).

### 2.3. Bacteria Culture and Identification

To study intratumour microbiota in epithelial ovarian tumours, tissue pieces (around 0.25 g) were homogenised with a glass homogeniser in 1 mL ice-cold PBS under sterile conditions. PBS was used as a tissue surrogate and the same workflow was performed to evaluate the environmental contaminants. Sample homogenates were placed on TSB (tryptic soy broth), MRS (Man Rogosa Sharpe), Cause’s synthetic agar medium, and BHI (brain heart infusion) solid medium. The bacteria were cultured continuously at 37 °C anaerobically and aerobically for 48–72 h. The number of bacteria in tumour tissue was approximately 4 × 10^4^ equivalent bacteria/gram tissue. To identify bacteria strain, colonies were picked and streaked in designated plates and conditions for 1–3 days to get single colonies, and subsequently the single colonies were sent to Sangon Biotech (Guangzhou, China) for sequencing. The forward primers were 5′-AGRGTTTGATCMTGGCTCAG-3′ and the reverse primers were 5′-TACGGYTACCTTGTTAYGACTT-3′. 

### 2.4. Establishment and Evaluation of Mouse EOC Model

The mouse ovarian epithelial cancer ID8 cell line was purchased from the Cell Bank of Fuxiang Biological (Jiaxing, China) and cultured in DMEM medium (Sangon Biotech) containing 10% foetal bovine serum (Sangon Biotech) and 100 U/mL penicillin/streptomycin (Solarbio, China), and were grown in a 37 °C incubator containing 5% CO_2_. Female C57BL/6 mice aged 5 weeks, provided by Hunan SJA Laboratory Animal Co. (Changsha, China), were raised in an ideal environment for one week (12 h light/dark cycle with ad libitum access to standard laboratory chow and water, humidity 50 ± 15%, temperature 22 ± 2 °C) before the subcutaneous injection of ID8 cells (5 × 10^6^ cells/mouse) on the mid-right flank to grow tumours. This study and the animal experiment protocol were viewed and approved by the Laboratory Animal Ethics Committee of Nanchang Royo Biotechnology Co, Ltd., Nanchang, China (approval number: RYE2021050701)

In order to evaluate the influence of endogenous mixed bacteria on the mouse EOC model, 40 mice were casually divided into four groups and treated as follows: (1) M group (*n* = 10): EOC model; (2) M-A group (*n* = 10): mixed antibiotics (metronidazole 1 g, neomycin 1 g, ampicillin 1 g, vancomycin 0.5 g) dissolved in 1 L of water for free drinking of mice were administered 2 weeks before tumour inoculation; (3) M-A-BMBT group (*n* = 10): mixed antibiotics were administered 2 weeks before tumour inoculation and intratumoural injection of 4 × 10^4^ CFU mixed bacteria from EBOT; (4) M-A-MMBT group (*n* = 10): mixed antibiotics were administered 2 weeks before tumour inoculation and 4 × 10^4^ CFU mixed bacteria from EOC was injected intratumourally. All treatments were given 2 weeks after tumour inoculation, twice a week for 4 weeks. The weight of the mice was recorded with a weight scale and the size of tumours were measured using callipers twice per week. At day 70, four mice of each group were euthanised and their tumours were removed and stored at −80 °C. The remaining mice were used to assess growth curves of tumours and survival. Tumour volume = length × width^2^ × 0.5 (repeat three times).

To further evaluate the effect of intratumour microbiota (mixed bacteria and *P. acnes* strain) on the mouse EOC model, 40 mice were erratically divided into four groups and treated as follows: (1) M group (*n* = 10): EOC model; (2) M-A group (*n* = 10): mixed antibiotics were administered 2 weeks before tumour inoculation; (3) M-A-SBT group (*n* = 10): mixed antibiotics were administered 2 weeks before tumour inoculation and intratumoural injection of 4 × 10^4^ CFU *P. acnes* strain; (4) M-A-MBT group (*n* = 10): mixed antibiotics were administered 2 weeks before tumour inoculation and 4 × 10^4^ CFU of mixed bacteria from EOC was injected intratumourally. The mixed bacteria and *P. acnes* strain injected intratumourally were given 2 weeks after tumour inoculation, twice a week for 4 weeks. At day 65, the procedures of tumour tissue removal, sample preparation, and the calculation of tumour growth curves and survival rates were as mentioned before.

To further confirm the specific molecular mechanism of *P. acnes* strain on the mouse EOC model, 70 mice were randomly divided into seven groups and treated as follows: (1) M group (*n* = 10): EOC model; (2) M-A group (*n* = 10): mixed antibiotics were administered 2 weeks before tumour inoculation; (3) M-A-SBT group (*n* = 10): mixed antibiotics were administered 2 weeks before tumour inoculation and intratumoural injection of 4 × 10^4^ CFU *P. acnes* strain; (4) M-A-MBT group (*n* = 10): mixed antibiotics were administered 2 weeks before tumour inoculation and 4 × 10 ^4^ CFU of mixed bacteria from EOC was injected intratumourally; (5) M-A-G group (*n* = 10): mixed antibiotics were administered 2 weeks before tumour inoculation and GANT61 (40 mg/kg) was dissolved in corn oil and subcutaneously injected; (6) M-A-MBT-G group (*n* = 10): mixed antibiotics were administered 2 weeks before tumour inoculation and 4 × 10^4^ CFU of mixed bacteria from EOC was injected intratumourally; (7) M-A-SBT-G group (*n* = 10): mixed antibiotics were administered 2 weeks before tumour inoculation and intratumoural injection of 4 × 10^4^ CFU *P. acnes* strain. The mixed bacteria and *P. acnes* strain were injected intratumourally 2 weeks after tumour inoculation, twice a week for 4 weeks, and GANT61 was injected subcutaneously 6 weeks after tumour inoculation at three times a week. At day 82, the procedures of tumour tissue removal, sample preparation, and the calculation for tumour growth curves and survival rates were as mentioned before. Specific chemical information and the various groups of mice with their respective characteristics and treatments is shown in Supplementary Tables S2 and S3.

### 2.5. Hematoxylin-Eosin Staining (HE Staining) and Immunohistochemistry (IHC)

After the fixation in 10% buffered formalin, all removed tumours were sectioned into 4–6 μm thick pieces and baked to dry. The paraffin sections were dehydrated gradually with ethanol after two 10-min soaks in xylene for HE staining. Sections were then removed, placed in distilled water, stained for 5 min with haematoxylin, washed in water for 1 min, and finally differentiated with hydrochloric acid, ethanol, and eosin solution. Xylene was translucent for 10 min twice after gradient dehydration by ethanol and sealed with neutral gum. An optical microscope was used to study the abnormal morphology.

For IHC, the tumour sections underwent deparaffinisation and rehydration, and were treated in 3% hydrogen peroxide to inhibit endogenous peroxidase activity. By microwaving the sections in a 10 mM sodium citrate solution (pH = 6.0), antigen recovery was accomplished. After blocking the sections with 2.5% horse serum, the sections were then incubated with antibodies for an overnight period at 4 °C. Utilising a complicated system of avidin and biotin, antibody detection was accomplished (Vector Laboratories, Burlingame, CA, USA). Slides were stained with 3,3′ diaminobenzidine, washed, and counterstained with haematoxylin. After the dehydration, they were subsequently treated with xylene and mounted.

### 2.6. RNA Extraction and Quantitative PCR

Using the Prime Script RT Master Mix Reverse Transcription Kit, complementary first-strand cDNA synthesis was carried out following the quick extraction of total RNA from fresh tumour tissues using Trizol (Prime Script RT Master Mix; Ta Ka Ra Biotechnology). The expression of interleukin (IL)-1β, IL-6, and tumour necrosis factor (TNF)-α inflammatory factor indicators was then examined using the SYBR green technique and an ABI 7900HT rapid real-time PCR instrument. Supplementary Table S4 contains a list of the primers needed for these analyses. Finally, real-time qPCR procedures were carried out in triplicate using GAPDH as the internal control gene and analytical results were derived using the 2^−ΔΔCt^ method.

### 2.7. Western Blotting

A similar amount of protease inhibitor mixed with RIPA lysis buffer (Solarbio) was added after the tumour tissue was placed in the centrifuge tube. After tissue was homogenised on ice, the supernatant was collected by centrifuging it at 12,000× *g* for 10 min at 4 °C to determine the protein concentration. Cellular proteins were then separated using 10–12% gel electrophoresis (SDS-PAGE) and transferred to polyvinylidene fluoride (PVDF) membranes, which were then blocked with 5% dry milk-TBST (20 mM Tris-HCl (pH 7.6), 127 mM NaCl, 0.1% Tween 20) at ambient temperature for an hour. Following an overnight incubation at 4 °C with a primary antibody that had been adequately diluted, PVDF was then incubated for 60 min at room temperature with a secondary antibody that had been diluted with 1% dry milk-TBST. The following antibodies were used: rabbit anti- β-actin (β-actin), mouse anti-smoothened homolog (Smo), rabbit anti-sonic hedgehog homolog (Shh), rabbit anti-patched homolog 1 (Ptch1), rabbit anti-glioma associated oncogene 1 (Gli1), and rabbit anti-glioma associated oncogene 2 (Gli2). The information of each specific antibody is shown in Supplementary Table S5 and the uncropped blots are shown in Supplementary Figures S2 and S3. In addition, the uncropped blots and molecular weight markers are shown in Supplementary Figures S4 and S5.

### 2.8. Statistical Analysis

GraphPad Prism version 9.0 was used for data handling, analysis, and graphical displays (GraphPad Software, Inc., San Diego, CA. USA). Nonparametric tests, one-way or two-way ANOVA, and annotations according to the International Convention for Statistical Representation were used to establish the statistical significance.

## 3. Results

### 3.1. The Taxonomic Diversity of Intratumour Microbiota in EOC and EBOT Tissues

**Figure 1:**
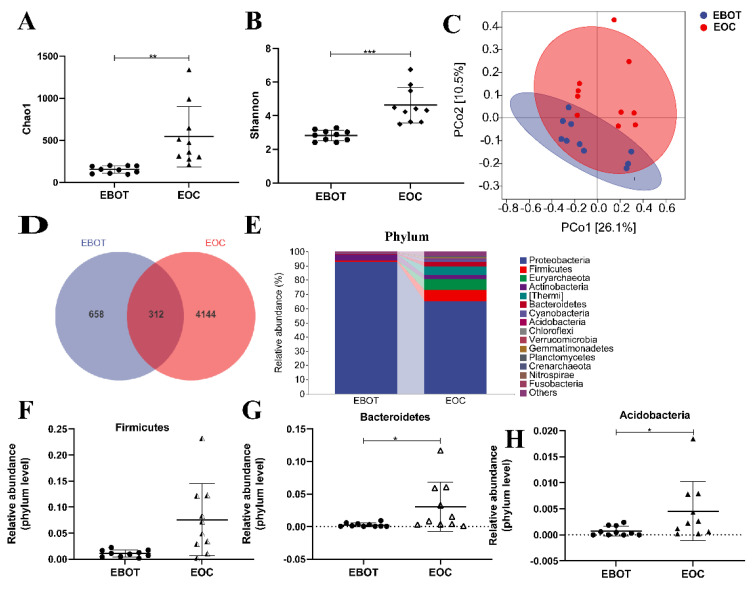
The microbial composition of EBOT and EOC tissues is distinct. (**A**) The Chao1 index. (**B**) The Shannon index. (**C**) PCoA of the weighted UniFrac distance demonstrated that the EBOT and EOC tissues showed two distinct clusters. (**D**) Venn map representation of OTUs. (**E**) Microbial composition in epithelial ovarian tumours at the phylum level. The relative abundance of (**F**) Firmicutes, (**G**) Bacteroidetes, and (**H**) Acidobacteria were analysed. Data are presented as means ± SD; * *p* < 0.05, ** *p* < 0.01, *** *p* < 0.001.

To characterise the intratumour microbiota among patients with EBOT and patients with EOC, 20 patients were enrolled in performing 16 s rRNA gene sequencing. Alpha diversity was analysed to identify differences in microbial diversity between the groups. The Chao1 index and Shannon index, which measures species richness and evenness, were significantly higher in EOC tissues than in EBOT tissue (541.6 vs. 151.4, *p* < 0.01 and 4.625 vs. 2.825, *p* < 0.001, respectively) (Figure 1A,B). Next, we examined beta diversity to compare the composition of EBOT and EOC tissues in the microbial community. The weighted UniFrac principal coordinate analysis (PCoA) revealed that clustering was observed across groups and the points of the EOC group were distant from the EBOT group in the plot, indicating that the EOC group’s microbial diversity was considerably different from that of the EBOT group (Figure 1C). According to the Venn diagram, 658 and 4144 OTUs were found in EOC and EBOT tissues, respectively, with 312 OTUs present in both categories (Figure 1D). Then, we further analysed the microbial composition at the phylum level and found that Firmicutes (1.077% and 7.567%), Bacteroidetes (0.2654% and 3.054%), and Acidobacteria (0.07428% and 0.45620%) were the dominant bacteria in EOC and EBOT tissues (Figure 1E–H).

### 3.2. Isolation of Intratumour Microbiota and Identification of Key Strains

**Figure 2:**
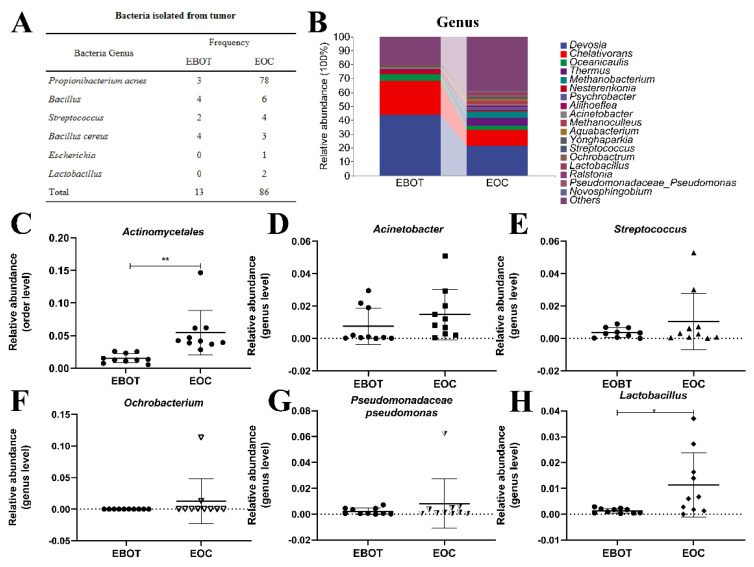
Isolation of intratumour microbiota and identification of key strains. (**A**) Table showing the culture isolated bacteria genera in EBOT and EOC. (**B**) Microbial composition in epithelial ovarian tumours at the order and genus level. The relative abundance of (**C**) *Actinomycetales*, (**D**) *Acinetobacter*, (**E**) *Streptococcus*, (**F**) *Ochrobacterium*, (**G**) *Pseudomonadaceae pseudomonas*, and (**H**) *Lactobacillus* were analysed. Data are presented as means ± SD; * *p* < 0.05, ** *p* < 0.01.

Since the intratumour microbiota of EOC has a higher alpha and beta diversity, the specific EOC-related bacteria were further isolated. We conducted bacterial culture and identification and then obtained 99 bacterial isolates, including 81 *P. acnes* isolates, 10 *Bacillus* isolates, 6 *Streptococcus* isolates, 7 *Bacillus cereus* isolates, 1 *Escherichia* isolate, and 2 *Lactobacillus* isolates. Specific strain information is shown in Figure 2A. As most bacterial isolates in the EOC were pathogenic bacteria, we wondered whether the bacterial isolation results are consistent with the sequencing results. Data from the top 18 microorganism populations were analysed at the order and genus level. As presented in Figure 2B, *Devosia*, *Chelativorans*, *Oceanicaulis*, and *Thermus* were the dominant genus in the EOC group. In addition, the abundance of previously considered pathogens *Actinomycetales*, *Acinetobacter*, *Streptococcus*, *Ochrobacterium*, and *Pseudomonadaceae Pseudomonas* was increased in the EOC tissues (Figure 2C–G), as well as *Lactobacillus*, which was viewed as a probiotic but may produce metabolites as an energy source for tumour growth and angiogenesis (Figure 2H).

### 3.3. Intratumour Microbiota in Human Epithelial Ovarian Carcinomas Facilitate EOC Progression in Mice

**Figure 3:**
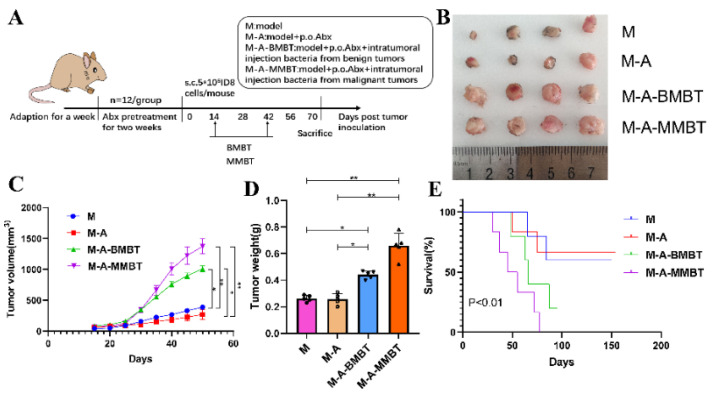
Injecting intratumourally with mixed bacteria from EOC significantly promoted tumour growth and reduced tumour model survival compared to mixed bacteria from EBOT. (**A**) Scheme of animal experiments. (**B**) Images of tumours in different treatment groups for 70 days. (**C**) Changes in tumour volume over time in different treatment groups. (**D**) Final tumour weights in different treatment groups. (**E**) Kaplan–Meier survival curves of mouse EOC models in different treatment groups. Data are presented as means ± SD. Two-way repeated measures ANOVA, both with Tukey’s test for multiple comparisons, (**B**–**D**), respectively, and log-rank test were performed for survival data (**E**); * *p* < 0.05, ** *p* < 0.01.

To further verify the tumourigenic effects of the intratumour microbiota in human epithelial ovarian carcinomas, a mouse EOC transplantation model was prepared, wherein mice of each group—except M group—were given mixed antibiotics dissolved in 1 L of water for free drinking for 2 weeks to eliminate as many microbes as possible from the intestines and tumours. Mice in different groups were injected intratumourally with intratumour microbiota extracted in the previous experiment (Figure 3A). Our results showed that injecting intratumourally with mixed bacteria from EOC and EBOT significantly increased the tumour volume compared to the M group (490.3 mm^3^ vs. 193.8 mm^3^, *p* < 0.05 and 611.2 mm^3^ vs. 193.8 mm^3^, *p* < 0.01, respectively) and the promoting effect of injecting intratumourally with mixed bacteria from EOC is more obvious than with mixed bacteria from EBOT (Figure 3B,C). Moreover, the tumour induced by injecting intratumourally with mixed bacteria from EOC and EBOT presented with a heavier tumour weight than group M (0.440 g vs. 0.262 g, *p* < 0.05 and 0.660 g vs. 0.262 g, *p* < 0.01, respectively) (Figure 3D). The survival test indicated that all mice injected intratumourally with mixed bacteria from EOC died before day 70, while mice injected intratumourally with mixed bacteria from EBOT and treated with antibiotics had survival rates of 33.3% and 66.7% on day 87, respectively (*p* < 0.01; Figure 3E).

### 3.4. Endogenous P. acnes May Promote EOC Progression in Mice via Regulating Hh Signalling Pathway

**Figure 4:**
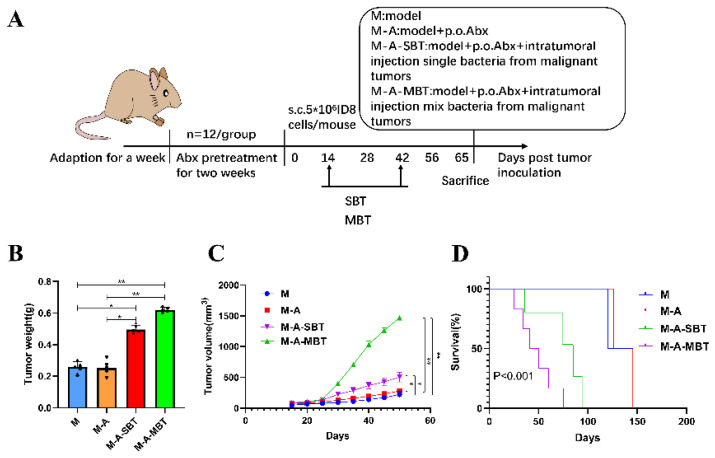
Injecting intratumourally with *P. acnes* from EOC can promote tumour growth and reduce tumour model survival as well as mixed bacteria from EOC. (**A**) Scheme of the animal experiment. (**B**) Changes in tumour volume over time in different treatment groups. (**C**) Final tumour weights in different treatment groups. (**D**) Kaplan–Meier survival curves of mouse EOC models in different treatment groups. Data are presented as means ± SD. Two-way repeated measures ANOVA, both with Tukey’s test for multiple comparisons, (**B**) and (**C**), respectively, and log-rank test were performed for survival data (**D**); * *p* < 0.05, ** *p* < 0.01.

To further determine the function of specific EOC-related bacteria in EOC progression, we then developed a mouse EOC model and provided the mice with mixed antibiotics through drinking water (DW) to efficiently eliminate both gut and tumour microbiota. The mixed bacteria and single bacteria (*P. acnes*) from EOC were injected intratumourally into the mice, respectively (Figure 4A). We found that injecting intratumourally with the *P. acnes* strain from EOC augmented the tumour volume compared with group M (264.0 mm^3^ vs. 115.9 mm^3^, *p* < 0.05, respectively), but the effect was not as evident as injecting intratumourally with mixed bacteria from EOC (650.7 mm^3^ vs. 115.9 mm^3^, *p* < 0.01, respectively) (Figure 4B). In addition, the tumour weight produced by injecting intratumourally with the *P. acnes* strain from EOC is heavier than group M (0.496 g vs. 0.258 g, *p* < 0.05, respectively) and the *P. acnes* strain shortened the survival rate of those mice compared with group M (33.3% vs. 66.7%, *p* < 0.05, respectively) (Figure 4C,D).

**Figure 5:**
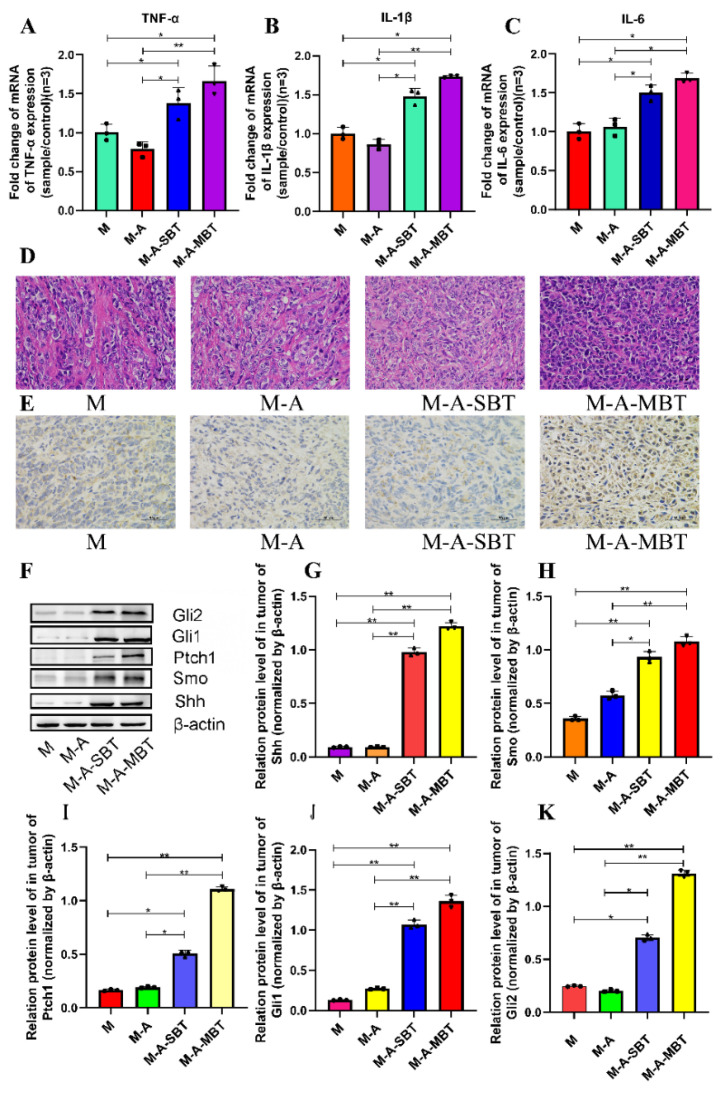
*P. acnes* promotes tumour growth by increasing the inflammatory response to activate the Hh signalling pathway. Proinflammatory cytokines (**A**) TNF-α, (**B**) IL-1β, and (**C**) IL-6 were detected in tumour tissues at the gene level by q-PCR. (**D**) H&E staining images of tumour tissues were presented; the bar represents 50 μm. (**E**) Immunohistochemistry of Gli1 expression was determined in EOC of mice; bar represents 50 μm. (**F**) Western blot analysis of Shh, Smo, Ptch1, Gli1, and Gli2 expression in tumour tissues; β-actin was used as an internal control (*n* = 3). The relative expressions of (**G**) Shh, (**H**) Smo, (**I**) Ptch1, (**J**) Gli1, and (**K**) Gli2 were quantified by Image J. Three mice were randomly selected in each group. Data are presented as means ± SD. One-way repeated measures ANOVA with Tukey’s test for multiple comparisons; * *p* < 0.05, ** *p* < 0.01.

To determine whether the tumour-promoting effect of the *P. acnes* strain is associated with tumour inflammation, the proinflammatory cytokines expressions were examined. As shown in Figure 5A–C, the mRNA levels of several proinflammatory cytokines increased significantly after treatment with the *P*. *acnes* strain on day 15, post-tumour inoculation. The tumour necrosis factor-α (TNF-α) level increased from 1.004 to 1.383 after injecting intratumourally with the P. acnes strain from EOC, and the value of interleukin-1β (IL-1β) was elevated from 1.002 to 1.481. All these results suggested that the tumour-promoting effect of the *P. acnes* strain may be associated with its proinflammatory property. Moreover, the H&E staining results showed that the *P. acnes* strain had increased the number of tumour cells. Compared to group M, the tumour cells were more homogeneous, thick, and deeply pigmented (Figure 5D). Since studies have shown that chronic inflammation can promote gastric cancer progression via Hh signalling, we wanted to further investigate whether inflammation caused by *P. acnes* can facilitate EOC progression through Hh signalling in mice. Therefore, key proteins in the Hh signalling pathway were further studied. The IHC results showed that the *P. acnes* strain significantly increased the expression of Gli1, whose high expression was thought to be associated with tumour progression (Figure 5E). In addition, we examined other key proteins in the Hh signalling pathway by Western blotting analysis. As shown in Figure 5, the *P. acnes* strain significantly enhanced the expression of Shh, Smo, Ptch1, Gli1, and Gli2 (*p* < 0.05; Figure 5F–K). In a word, the tumour-promoting effect of intratumour microbiota may be partially related to its increased inflammatory response to activate Hh signalling pathway.

### 3.5. Suppression of Hh Signalling Inhibited the EOC Progression Caused by P. acnes

**Figure 6:**
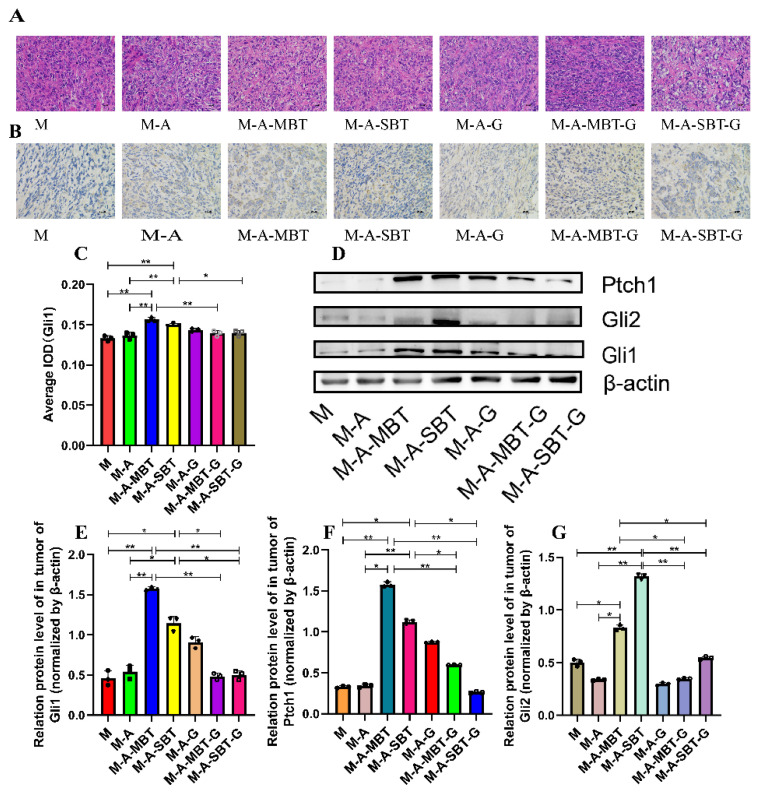
Suppression of Hh signalling proved that *P. acnes* promotes EOC progression in mice by regulating the Hh signalling pathway. (**A**) H&E staining images of tumour tissues were presented; bar represents 50 μm. (**B**) Immunohistochemistry of Gli1 expression was determined in ovarian cancer of mice; the bar represents 50 μm. (**C**) Average integrated optical density (IOD) value of Gli1 expression in each group of tumour specimens. (**D**) Western blot analysis of Ptch1, Gli1, and Gli2 expression in tumour tissues; β-actin was used as an internal control (*n* = 3). The relative expressions of (**E**) Ptch1, (**F**) Gli1, and (**G**) Gli2 were quantified by Image J. Data are presented as means ± SD. Two-way repeated-measures ANOVA, both with Tukey’s test for multiple comparisons; * *p* < 0.05, ** *p* < 0.01.

Previous results indicated that the tumour-promoting effect of *P. acnes* might be related to aberrant activation of Hh signalling. To further verify the tumour-promoting role of the Hh signalling pathway in EOC, we firstly examined the effects of GANT61 (the specific inhibitor of Gli1 and Gli2) by measuring the expression of Hh receptor, Ptch, and effectors, Gli1 and Gli2. The H&E staining results showed that GANT61 could increase necrotic tumour cells and enlarge the necrotic area to a greater extent than injecting intratumourally with the *P. acnes* strain from EOC (Figure 6A). The IHC results demonstrated that GANT61 usage significantly decreased Gli1 expression (Figure 6B,C). Additionally, GANT61 reduced the expression of the transcription factors Gli1 and Gli2, according to the outcomes of Western blotting (Figure 6D–F). Meanwhile, Ptch, a downstream target of Gli, was prevented from expressing by GANT61 (Figure 6G). These results suggested that the *P. acnes* tumour-promoting effect was associated with the activation of Hh signalling pathways.

## 4. Discussion

EOC is a common gynaecological malignancy originated from fallopian tube cancer and peritoneal malignancy which contributes to most of the mortalities in gynaecological tumours, yet with a still unsatisfactory clinical outcome [26,27]. Many recent studies have confirmed the existence of intratumour bacteria and elucidated their functions on tumour biology [28,29,30,31,32]. Research has indicated that *Peptostreptococcus anaerobius* is able to promote colonic tumourigenesis by influencing the PI3K-AKT pathway to regulate both proinflammatory and immunosuppressive responses [33]. Similarly, *pks + E. coli*, *Clostridium spp*., and *Bacteroides fragilis* in the tumour can also contribute to tumour formation and development [34,35]. However, there are few studies on the microbial composition of EBOT or EOC and the potential role of intratumour microbiota in EOC, and its molecular mechanism is still lacking.

In the present study, 20 patients were collected to characterise the intratumour microbiota among patients with EBOT and patients with EOC, based on the high-throughput sequencing method. We found that intratumour microbiota in EOC tissues has a higher diversity and richness compared to EBOT tissues, with Firmicutes, Bacteroidetes, and Acidobacteria being the dominant bacteria in both groups (Figure 1). Meanwhile, we found that *Actinomycetales*, *Acinetobacter*, *Streptococcus*, *Ochrobacterium*, and *Pseudomonadaceae Pseudomonas* exhibited higher abundance in the EOC tissues (Figure 2). Since the intratumour microbiota of EOC has a higher alpha and beta diversity, we further isolated the specific EOC-related bacteria and obtained 81 *P. acnes* isolates. Based on our previous research that *P. acnes* occurs with the highest frequency in EOC patients, we speculate that *P. acnes* is a key strain in facilitating EOC progression. *P. acnes* is a typical opportunistic bacterium commonly occurring on the skin, especially associated with acne vulgaris [36]. It is confirmed that *P. acnes* can induce intervertebral disc degeneration by promoting iNOS/NO and COX-2/PGE2 activation via the ROS-dependent NF-κB pathway, and recent studies have also highlighted its role in oncogenesis [37]. Reports on isolation of *P. acnes* from prostate cancer indicated a link between this microorganism and etiopathogenesis of hypertrophic prostatitis, suggesting that *P. acnes* promotes cell proliferation and malignant transformation [38]. This provided clues for revealing the role of intratumoural microbiota during the development of EOC.

Thus, based on the results above and previous findings that intratumoural microbiota contribute to the initiation and progression of cancer, we constructed a mouse EOC model to evaluate the effect of endogenous mixed bacteria on EOC. Mixed bacteria were injected intratumourally into the mouse EOC model, and the results showed that the mixed bacteria from EBOT and EOC are capable of promoting tumour growth and reducing the survival rate of mice, with the tumour-promoting effect of mixed bacteria from EOC proving more significant than mixed bacteria from EBOT (Figure 3). Furthermore, to evaluate the influence of *P. acnes* on EOC, mixed bacteria and the *P. acnes* strain were injected intratumourally into the mouse EOC model, respectively. As shown in Figure 4, we found that the *P. acnes* strain had a similar effect as mixed bacteria. These results confirmed the facilitating role of *P. acnes* in the progression of EOC.

We further explored the potential mechanisms of *P. acnes* function in EOC. Existing data showed that chronic inflammation is an important risk factor for EOC. It could activate inflammatory signalling pathways and immune responses, and subsequently promote the transformation of normal fallopian tube and ovarian cells into malignant cells [39,40]. Our data also suggested that *P. acnes*-treated EOC mice had an apparent intratumoural inflammatory condition, mainly characterised by increased expression of IL-1β, IL-6, and TNF-α. As IL-1β and TNF-α are two prominent proinflammatory cytokines excreted by necrotic tumour cells, some evidence indicated that proinflammatory cytokines of TNF-α/IL-1β could activate Hh pathway with a profile of the upregulated expression of Gli1 and Shh expression, thus they are able to directly reflect the degree of tumour necrosis through the stimulation of Hh transcription [41]. The Hh signalling pathway is a cascade involving Hh, Ptch, Smo, and Gli which regulate diverse processes ranging from tissue patterning and cell differentiation to cancer initiation, progression, and metastasis [42]. Aberrant Hh signalling activation is associated with various diseases, including basal cell carcinoma (BCC), sporadic medulloblastoma, and ovarian cancer [43,44]. For example, the overexpression of Gli1, a key protein in the Hh pathway, is considered as a symbol of Hh pathway activation and is able to activate oncogene target genes such as Cyclin-D1, Myc, Bcl-2, Ang1/2, Snai1, Nanog, and Sox2 to promote carcinogenesis and tumour development [43]. In this study, we demonstrated through IHC analysis that Gli1 overexpression could be induced by *P. acnes*, and this species was also proven to be associated with the upregulated levels of other key proteins in the Hh signalling pathway, including Shh, Smo, Ptch1, and Gli2 (Figure 5), suggesting that the Hh signalling pathway was abnormally activated in EOC mice. Research by Zavros manifested that *H. pylori* infection had a similar effect by utilising the Hh pathway, which also increased the protein expression level in the parietal cells [45]. Collectively, the tumour-promoting effect of the *P. acnes* strain may be partially related to its increased inflammatory response to activate Hh signalling pathway.

To confirm our previous results, we injected GANT61 into the mouse EOC model and discovered that tumour growth was visibly reduced, and survival time was significantly increased. Moreover, protein analysis showed that Gli1, Gli2, and Ptch1 were significantly downregulated in the Hh signalling pathway. IHC analysis of Gli1 was consistent with the previous trend, which further confirmed the key molecular mechanism that microbial-induced inflammation promotes EOC progression through Hh signalling (Figure 6).

In summary, we first profiled the intratumour microbiota of EBOT and EOC tissues and demonstrated that the diversity and composition of the intratumour microbiota were significantly different. More importantly, through in vivo and in vitro experiments, we confirmed the molecular mechanism of intratumour microbiota promotion of EOC progression in mice, which induces inflammation to activate the Hh signalling pathway. However, our study still had several shortcomings. First, the sample size was small, which accounts for the lack of a statistically significant association between clinical characteristics and microbiota. Second, in the process of collecting samples, we could not completely eliminate the possibility of contamination. At last, we failed to screen several bacteria with a high relative abundance, possibly due to our limited screening conditions. Therefore, future studies are required for further in-depth analysis of how the bacteria invade tumour cells, how the intracellular bacteria are integrated into the host–cell system, and how the bacteria-containing tumour cells interact with the immune system, which will provide us insights on improving EOC therapeutics.

## 5. Conclusions

This study indicated that the microbial composition of EBOT and EOC tissues is distinct. In addition, we found that *P. acnes* is a key strain in facilitating EOC progression and discovered the underlying molecular mechanisms, which were associated with the increased inflammatory response to activate the Hh signalling pathway. This could provide us clues for improving EOC treatment.

## Data Availability

Sequencing data associated with this study have been deposited in the NCBI repository, BioProject ID PRJNA876390.

## Supplementary Materials

The following supporting information can be downloaded at: https://www.mdpi.com/article/10.3390/cancers14215178/s1, Figure S1: This study initially included 30 patients, of which 3 were excluded due to chemotherapy treatment and severe comorbidity. Thereafter, 27 patients were collected for bacterial and microbial DNA extraction. During this process, 7 patients failed to extract DNA and were excluded; Figure S2. Original Western blot images for Figure 5F; Figure S3. Original Western blot images for Figure 6D; Figure S4: The uncropped blots and molecular weight markers for Figure 5F; Figure S5: The uncropped blots and molecular weight markers for Figure 6D; Table S1: Characteristics of patients with epithelial ovarian tumour; Table S2: Chemicals information; Table S3: The grouping treatment of the animals; Table S4: The information of primers; Table S5: The information of each specific antibody.

## References

[1] Song M., Yeku O.O., Rafiq S., Purdon T., Dong X., Zhu L., Zhang T., Wang H., Yu Z., Mai J. (2020). Tumor derived UBR5 promotes ovarian cancer growth and metastasis through inducing immunosuppressive macrophages. Nat. Commun..

[2] Daniels M.S., Babb S.A., King R.H., Urbauer D.L., Batte B.A., Brandt A.C., Amos C.I., Buchanan A.H., Mutch D.G., Lu K.H. (2014). Underestimation of risk of a BRCA1 or BRCA2 mutation in women with high-grade serous ovarian cancer by BRCAPRO: A multi-institution study. J. Clin. Oncol. Off. J. Am. Soc. Clin. Oncol..

[3] Karakashev S., Fukumoto T., Zhao B., Lin J., Wu S., Fatkhutdinov N., Park P.H., Semenova G., Jean S., Cadungog M.G. (2020). EZH2 Inhibition Sensitizes CARM1-High, Homologous Recombination Proficient Ovarian Cancers to PARP Inhibition. Cancer Cell.

[4] Krzystyniak J., Ceppi L., Dizon D.S., Birrer M.J. (2016). Epithelial ovarian cancer: The molecular genetics of epithelial ovarian cancer. Ann. Oncol. Off. J. Eur. Soc. Med. Oncol..

[5] Gallagher D.J., Konner J.A., Bell-McGuinn K.M., Bhatia J., Sabbatini P., Aghajanian C.A., Offit K., Barakat R.R., Spriggs D.R., Kauff N.D. (2011). Survival in epithelial ovarian cancer: A multivariate analysis incorporating BRCA mutation status and platinum sensitivity. Ann. Oncol. Off. J. Eur. Soc. Med. Oncol..

[6] Vergote I.B., Garcia A., Micha J., Pippitt C., Bendell J., Spitz D., Reed N., Dark G., Fracasso P.M., Ibrahim E.N. (2013). Randomized multicenter phase II trial comparing two schedules of etirinotecan pegol (NKTR-102) in women with recurrent platinum-resistant/refractory epithelial ovarian cancer. J. Clin. Oncol. Off. J. Am. Soc. Clin. Oncol..

[7] Banerjee S., Rustin G., Paul J., Williams C., Pledge S., Gabra H., Skailes G., Lamont A., Hindley A., Goss G. (2013). A multicenter, randomized trial of flat dosing versus intrapatient dose escalation of single-agent carboplatin as first-line chemotherapy for advanced ovarian cancer: An SGCTG (SCOTROC 4) and ANZGOG study on behalf of GCIG. Ann. Oncol. Off. J. Eur. Soc. Med. Oncol..

[8] Mitra R., Chen X., Greenawalt E.J., Maulik U., Jiang W., Zhao Z., Eischen C.M. (2017). Decoding critical long non-coding RNA in ovarian cancer epithelial-to-mesenchymal transition. Nat. Commun..

[9] Uddin S., Bu R., Ahmed M., Abubaker J., Al-Dayel F., Bavi P., Al-Kuraya K.S. (2009). Overexpression of leptin receptor predicts an unfavorable outcome in Middle Eastern ovarian cancer. Mol. Cancer.

[10] Li X., Hu Z., Shi H., Wang C., Lei J., Cheng Y. (2020). Inhibition of VEGFA Increases the Sensitivity of Ovarian Cancer Cells to Chemotherapy by Suppressing VEGFA-Mediated Autophagy. OncoTargets Ther..

[11] Morita T., Mayanagi T., Sobue K. (2007). Dual roles of myocardin-related transcription factors in epithelial mesenchymal transition via slug induction and actin remodeling. J. Cell Biol..

[12] Louro I.D., Bailey E.C., Li X., South L.S., McKie-Bell P.R., Yoder B.K., Huang C.C., Johnson M.R., Hill A.E., Johnson R.L. (2002). Comparative gene expression profile analysis of GLI and c-MYC in an epithelial model of malignant transformation. Cancer Res..

[13] Zeng C., Chen T., Zhang Y., Chen Q. (2017). Hedgehog signaling pathway regulates ovarian cancer invasion and migration via adhesion molecule CD24. J. Cancer.

[14] Zhang H., Wang Y., Chen T., Zhang Y., Xu R., Wang W., Cheng M., Chen Q. (2019). Aberrant Activation Of Hedgehog Signalling Promotes Cell Migration And Invasion Via Matrix Metalloproteinase-7 In Ovarian Cancer Cells. J. Cancer.

[15] Juarez V.M., Montalbine A.N., Singh A. (2022). Microbiome as an immune regulator in health, disease, and therapeutics. Adv. Drug Deliv. Rev..

[16] Cho I., Blaser M.J. (2012). The human microbiome: At the interface of health and disease. Nat. Rev. Genet..

[17] Gérard P. (2016). Gut microbiota and obesity. Cell. Mol. Life Sci. CMLS.

[18] Sun J., Kato I. (2016). Gut microbiota, inflammation and colorectal cancer. Genes Dis..

[19] Huang H., Ren Z., Gao X., Hu X., Zhou Y., Jiang J., Lu H., Yin S., Ji J., Zhou L. (2020). Integrated analysis of microbiome and host transcriptome reveals correlations between gut microbiota and clinical outcomes in HBV-related hepatocellular carcinoma. Genome Med..

[20] Flemer B., Lynch D.B., Brown J.M., Jeffery I.B., Ryan F.J., Claesson M.J., O’Riordain M., Shanahan F., O’Toole P.W. (2017). Tumour-associated and non-tumour-associated microbiota in colorectal cancer. Gut.

[21] Urbaniak C., Gloor G.B., Brackstone M., Scott L., Tangney M., Reid G. (2016). The Microbiota of Breast Tissue and Its Association with Breast Cancer. Appl. Environ. Microbiol..

[22] Riquelme E., Zhang Y., Zhang L., Montiel M., Zoltan M., Dong W., Quesada P., Sahin I., Chandra V., San Lucas A. (2019). Tumor Microbiome Diversity and Composition Influence Pancreatic Cancer Outcomes. Cell.

[23] Nejman D., Livyatan I., Fuks G., Gavert N., Zwang Y., Geller L.T., Rotter-Maskowitz A., Weiser R., Mallel G., Gigi E. (2020). The human tumor microbiome is composed of tumor type-specific intracellular bacteria. Science.

[24] Geller L.T., Barzily-Rokni M., Danino T., Jonas O.H., Shental N., Nejman D., Gavert N., Zwang Y., Cooper Z.A., Shee K. (2017). Potential role of intratumor bacteria in mediating tumor resistance to the chemotherapeutic drug gemcitabine. Science.

[25] Fu A., Yao B., Dong T., Chen Y., Yao J., Liu Y., Li H., Bai H., Liu X., Zhang Y. (2022). Tumor-resident intracellular microbiota promotes metastatic colonization in breast cancer. Cell.

[26] Ducie J., Dao F., Considine M., Olvera N., Shaw P.A., Kurman R.J., Shih I.M., Soslow R.A., Cope L., Levine D.A. (2017). Molecular analysis of high-grade serous ovarian carcinoma with and without associated serous tubal intra-epithelial carcinoma. Nat. Commun..

[27] Del Campo J.M., Matulonis U.A., Malander S., Provencher D., Mahner S., Follana P., Waters J., Berek J.S., Woie K., Oza A.M. (2019). Niraparib Maintenance Therapy in Patients With Recurrent Ovarian Cancer After a Partial Response to the Last Platinum-Based Chemotherapy in the ENGOT-OV16/NOVA Trial. J. Clin. Oncol. Off. J. Am. Soc. Clin. Oncol..

[28] Banerjee S., Wei Z., Tan F., Peck K.N., Shih N., Feldman M., Rebbeck T.R., Alwine J.C., Robertson E.S. (2015). Distinct microbiological signatures associated with triple negative breast cancer. Sci. Rep..

[29] Banerjee S., Tian T., Wei Z., Shih N., Feldman M.D., Peck K.N., DeMichele A.M., Alwine J.C., Robertson E.S. (2018). Distinct Microbial Signatures Associated With Different Breast Cancer Types. Front. Microbiol..

[30] Jin C., Lagoudas G.K., Zhao C., Bullman S., Bhutkar A., Hu B., Ameh S., Sandel D., Liang X.S., Mazzilli S. (2019). Commensal Microbiota Promote Lung Cancer Development via γδ T Cells. Cell.

[31] Xuan C., Shamonki J.M., Chung A., Dinome M.L., Chung M., Sieling P.A., Lee D.J. (2014). Microbial dysbiosis is associated with human breast cancer. PLoS ONE.

[32] Wegiel B., Vuerich M., Daneshmandi S., Seth P. (2018). Metabolic Switch in the Tumor Microenvironment Determines Immune Responses to Anti-cancer Therapy. Front. Oncol..

[33] Gao Z., Guo B., Gao R., Zhu Q., Qin H. (2015). Microbiota disbiosis is associated with colorectal cancer. Front. Microbiol..

[34] Osman M.A., Neoh H.M., Ab Mutalib N.S., Chin S.F., Mazlan L., Raja Ali R.A., Zakaria A.D., Ngiu C.S., Ang M.Y., Jamal R. (2021). Parvimonas micra, Peptostreptococcus stomatis, Fusobacterium nucleatum and Akkermansia muciniphila as a four-bacteria biomarker panel of colorectal cancer. Sci. Rep..

[35] Baxter N.T., Zackular J.P., Chen G.Y., Schloss P.D. (2014). Structure of the gut microbiome following colonization with human feces determines colonic tumor burden. Microbiome.

[36] Olender A., Radej S., Płaza P., Bar K., Maciejewski R. (2016). Propionibacterium acnes infection associated with cancerous prostate hypertrophy. Pol. Arch. Med. Wewn..

[37] Bae Y., Ito T., Iida T., Uchida K., Sekine M., Nakajima Y., Kumagai J., Yokoyama T., Kawachi H., Akashi T. (2014). Intracellular Propionibacterium acnes infection in glandular epithelium and stromal macrophages of the prostate with or without cancer. PLoS ONE.

[38] Ugge H., Udumyan R., Carlsson J., Andrén O., Montgomery S., Davidsson S., Fall K. (2018). Acne in late adolescence and risk of prostate cancer. Int. J. Cancer.

[39] Jia D., Nagaoka Y., Katsumata M., Orsulic S. (2018). Inflammation is a key contributor to ovarian cancer cell seeding. Sci. Rep..

[40] Sapoznik S., Bahar-Shany K., Brand H., Pinto Y., Gabay O., Glick-Saar E., Dor C., Zadok O., Barshack I., Zundelevich A. (2016). Activation-Induced Cytidine Deaminase Links Ovulation-Induced Inflammation and Serous Carcinogenesis. Neoplasia.

[41] Kim J.E., Phan T.X., Nguyen V.H., Dinh-Vu H.V., Zheng J.H., Yun M., Park S.G., Hong Y., Choy H.E., Szardenings M. (2015). Salmonella typhimurium Suppresses Tumor Growth via the Pro-Inflammatory Cytokine Interleukin-1β. Theranostics.

[42] He S., Wang L., Miao L., Wang T., Du F., Zhao L., Wang X. (2009). Receptor interacting protein kinase-3 determines cellular necrotic response to TNF-alpha. Cell.

[43] Chaudhry P., Singh M., Triche T.J., Guzman M., Merchant A.A. (2017). GLI3 repressor determines Hedgehog pathway activation and is required for response to SMO antagonist glasdegib in AML. Blood.

[44] Qian H., Cao P., Hu M., Gao S., Yan N., Gong X. (2019). Inhibition of tetrameric Patched1 by Sonic Hedgehog through an asymmetric paradigm. Nat. Commun..

[45] Merchant J.L., Ding L. (2017). Hedgehog Signaling Links Chronic Inflammation to Gastric Cancer Precursor Lesions. Cell. Mol. Gastroenterol. Hepatol..

