# Correction: Huang et al. Endogenous Propionibacterium acnes Promotes Ovarian Cancer Progression via Regulating Hedgehog Signalling Pathway. *Cancers* 2022, *14*, 5178

**DOI:** 10.3390/cancers16183185

**Published:** 2024-09-18

**Authors:** Qifa Huang, Xin Wei, Wenyu Li, Yanbing Ma, Guanxiang Chen, Lu Zhao, Ying Jiang, Siqi Xie, Qi Chen, Tingtao Chen

**Affiliations:** 1 Department of Obstetrics & Gynecology, The Second Affiliated Hospital of Nanchang University, Nanchang 330006, China; 2 National Engineering Research Center for Bioengineering Drugs and the Technologies, Institute of Translational Medicine, Nanchang University, Nanchang 330031, China

In the original publication [1], there was a mistake in the legend for Figure 6. We apologise for the error in our manuscript and legend, which claimed that Figure 6 included H–K images when, in fact, only A–G images were present. This oversight was regrettable, and I apologise once again for any resulting confusion. The correct legend appears below.

**Figure 6.** Suppression of Hh signalling proved that *P. acnes* promotes EOC progression in mice by regulating the Hh signalling pathway. (**A**) H&E staining images of tumour tissues were presented; bar represents 50 μm. (**B**) Immunohistochemistry of Gli1 expression was determined in ovarian cancer of mice; the bar represents 50 μm. (**C**) Average integrated optical density (IOD) value of Gli1 expression in each group of tumour specimens. (**D**) Western blot analysis of Ptch1, Gli1, and Gli2 expression in tumour tissues; β-actin was used as an internal control (*n* = 3). The relative expressions of (**E**) Ptch1, (**F**) Gli1, and (**G**) Gli2 were quantified by Image J. Data are presented as means ± SD. Two-way repeated-measures ANOVA, both with Tukey’s test for multiple comparisons; * *p* < 0.05, ** *p* < 0.01.

## Error in Table

In the original publication, there was a mistake in Table S1 as published. As you know, when patients suspected of having ovarian tumours are admitted, they undergo a series of biochemical and imaging examinations before surgical evaluation, which typically takes some time. So, relying solely on the initial imaging diagnosis upon admission does not meet our formal recruitment criteria, and we also needed a clear patient type to ensure the accuracy of our sample. Therefore, firstly, we selected 15 patients in each group with the most possibility to be our candidate when they were in the hospital, and then selected 10 patients engaged in our group, whose results are indicative and require confirmation through postoperative pathological examination combined with relevant imaging and biochemical results to make a definitive diagnosis. So, only 10 patients were enrolled on our work, and the initial mention of 15 patients per group was merely a preliminary screening number and did not meet our final inclusion criteria. This method aligns with the ethical standards outlined in our application and ensures compliance with clinical trial ethics requirements. The corrected Table S1 appears below.

**Table S1:** Characteristics of patients with epithelial ovarian tumour.

Characteristics	EBOT	EOC
Total number	10	10
Gender(no.)		
Female	10	10
Male	0	0
Age (years, mean ± SD)	39.00 ± 3.70	50.80 ± 4.40
Weight (kg, mean ± SD)	55.50 ± 1.54	53.20 ± 1.52
BMI (mean ± SD)	20.52 ± 0.36	20.01 ± 0.27

There was an error in the original publication. As previously mentioned, to ensure the successful enrollment of 10 patients in each of the remaining two groups, we initially screened 15 patients in each group based on imaging examination results. Only those who passed the initial screening were considered for enrollment. Subsequently, following postoperative pathological diagnosis and relevant biochemical tests, we enrolled 10 patients in each group to align with our ethical guidelines. We are sorry for the number of 15 in our introduction, method, results and discussion part, as the students did not clearly distinguish the difference between initial screening and final enrollment. However, as you can see in Figure 1, the number is only 10 in our work (one dots mean one patient, and there are only ten dots in each group). In addition, I apologise for any confusion caused by the registration number issue. It appears that we inadvertently used a template from the China Clinical Trial Registry and copied its registration number without updating it prior to submission. The correct registration number for our study is ChiCTR2000041320. We will ensure that this correction is made to the manuscript. Furthermore, we apologise for the oversight in duplicating the sentence under the “2. Materials and Methods” section. Our carelessness in writing the article has led to the inadvertent repetition here. We are willing to conduct a thorough review of the article’s content to address and rectify this error.

A correction has been made to Abstract:

Background: The oncogenesis and progression of epithelial ovarian cancer (EOC) is a complicated process involving several key molecules and factors, yet whether microbiota are present in EOC, and their role in the development of EOC, remains greatly unknown. Methods: In this study, 20 patients were enrolled to compare the similarities and differences of intratumour microbiota among patients with epithelial benign ovarian tumours (EBOTs) and patients with EOC based on the high-throughput sequencing method. Subsequently, we further isolated the specific EOC-related bacteria and defined *Propionibacterium acnes* as a key strain in facilitating EOC progression. More importantly, we constructed a mouse EOC model to evaluate the effect of the *P. acnes* strain on EOC using immunohistochemistry, Western blotting, and RT-qPCR. Results: The high-throughput sequencing showed that the intratumour microbiota in EOC tissues had a higher microbial diversity and richness compared to EBOT tissues. The abundance of previously considered pathogens, *Actinomycetales*, *Acinetobacter*, *Streptococcus*, *Ochrobacterium*, and *Pseudomonadaceae Pseudomonas*, was increased in the EOC tissues. Meanwhile, we discovered the facilitating role of the *P. acnes* strain in the progression of EOC, which may be partially associated with the increased inflammatory response to activate the hedgehog (Hh) signalling pathway. This microbial-induced EOC progression mechanism is further confirmed using the inhibitor GANT61. Conclusions: This study profiled the intratumour microbiota of EBOT and EOC tissues and demonstrated that the diversity and composition of the intratumour microbiota were significantly different. Furthermore, through in vivo and in vitro experiments, we confirmed the molecular mechanism of intratumour microbiota promotion of EOC progression in mice, which induces inflammation to activate the Hh signalling pathway. This could provide us clues for improving EOC treatment.

A correction has been made to 1. Introduction, Paragraph 4:

In this study, 20 patients were enrolled to characterise the intratumour microbiota among patients with EBOT (Average age: 39.00, *n* = 10) and patients with EOC (Average age: 50.80, *n* = 10) based on the 16s rRNA gene sequencing method. Moreover, bacterial culture and identification were conducted to isolate the specific EOC-related bacteria, which was then intratumourally injected into ID8 cells induced mouse EOC models. The protumourigenic effects of this strain in mice were verified using immunohistochemistry, Western blotting, and RT-qPCR to further ascertain the underlying molecular mechanisms, which could be viewed as strong implications for future EOC treatment.

A correction has been made to 2. Materials and Methods, 2.1. Enrollment of Patients with EOC and Collection of Tumour Samples, Paragraphs 1 and 2:

Tumour tissue samples and clinical data were collected from 20 patients with epithelial ovarian tumours admitted from November 2020 to December 2021 (Figure S1). Inclusion criteria were as follows: (1) The patients were aged 18–75 years old and all patients with epithelial ovarian tumours (serous tumour, mucinous tumour) were indicated by imaging and confirmed by postoperative pathology; (2) All patients were in good mental condition, with no change in weight and normal vital signs such as heart rate, breathing, and blood pressure, did not use antibiotics within the past three months, and denied severe heart, lung, kidney, or liver dysfunction and metabolic diseases; (3) Patients with severe comorbidity and previous radiotherapy or chemotherapy treatment were excluded. General anaesthesia was performed for all patients before operation, most of whom received radical resection of epithelial ovarian tumours. Fresh tissues of EBOT and EOC were collected in the sterile surgery room from the Second Affiliated Hospital of Nanchang University and were immediately transferred to sterile 15 mL conical tubes with sterile DMEM culture medium. Samples were processed in the clean and sterile cell culture hood with autoclaved dissection tools, as much as possible, to reduce contamination. Characteristics of patients with epithelial ovarian tumour is shown in Supplementary Table S1.

Patients were divided into EBOT group (ovarian pathological biopsy suggests serous cystadenoma or myxoid cystadenoma, *n* = 10) and EOC group (ovarian pathological biopsy suggests serous cystadenocarcinoma or myxoid cystadenocarcinoma, *n* = 10). All patients in this clinical study were informed in advance, with consent signed before the tumour tissue collection. This study was approved by the ethics committee of the Second Affiliated Hospital of Nanchang University (IRB-2020-061) and registered by the China Clinical Trial Registry (no. ChiCTR2000041320).

A correction has been made to 3. Results, 3.1. The Taxonomic Diversity of Intratumour Microbiota in EOC and EBOT Tissues, Paragraph 1:

To characterise the intratumour microbiota among patients with EBOT and patients with EOC, 20 patients were enrolled in performing 16 s rRNA gene sequencing. Alpha diversity was analysed to identify differences in microbial diversity between the groups. The Chao1 index and Shannon index, which measures species richness and evenness, were significantly higher in EOC tissues than in EBOT tissue (541.6 vs. 151.4, *p* < 0.01 and 4.625 vs. 2.825, *p* < 0.001, respectively) (Figure 1A,B). Next, we examined beta diversity to compare the composition of EBOT and EOC tissues in the microbial community. The weighted UniFrac principal coordinate analysis (PCoA) revealed that clustering was observed across groups and the points of the EOC group were distant from the EBOT group in the plot, indicating that the EOC group’s microbial diversity was considerably different from that of the EBOT group (Figure 1C). According to the Venn diagram, 658 and 4144 OTUs were found in EOC and EBOT tissues, respectively, with 312 OTUs present in both categories (Figure 1D). Then, we further analysed the microbial composition at the phylum level and found that Firmicutes (1.077% and 7.567%), Bacteroidetes (0.2654% and 3.054%), and Acidobacteria (0.07428% and 0.45620%) were the dominant bacteria in EOC and EBOT tissues (Figure 1E–H).

A correction has been made to 3. Results, 3.5. Suppression of Hh Signalling Inhibited the EOC Progression Caused by *P. acnes*, Paragraph 1:

Previous results indicated that the tumour-promoting effect of *P. acnes* might be related to aberrant activation of Hh signalling. To further verify the tumour-promoting role of the Hh signalling pathway in EOC, we firstly examined the effects of GANT61 (the specific inhibitor of Gli1 and Gli2) by measuring the expression of Hh receptor, Ptch, and effectors, Gli1 and Gli2. The H&E staining results showed that GANT61 could increase necrotic tumour cells and enlarge the necrotic area to a greater extent than injecting intratumourally with the *P. acnes* strain from EOC (Figure 6A). The IHC results demonstrated that GANT61 usage significantly decreased Gli1 expression (Figure 6B,C). Additionally, GANT61 reduced the expression of the transcription factors Gli1 and Gli2, according to the outcomes of Western blotting (Figure 6D–F). Meanwhile, Ptch, a downstream target of Gli, was prevented from expressing by GANT61 (Figure 6G). These results suggested that the *P. acnes* tumour-promoting effect was associated with the activation of Hh signalling pathways.

A correction has been made to 4. Discussion, Paragraph 2:

In the present study, 20 patients were collected to characterise the intratumour microbiota among patients with EBOT and patients with EOC, based on the high-throughput sequencing method. We found that intratumour microbiota in EOC tissues has a higher diversity and richness compared to EBOT tissues, with Firmicutes, Bacteroidetes, and Acidobacteria being the dominant bacteria in both groups (Figure 1). Meanwhile, we found that *Actinomycetales*, *Acinetobacter*, *Streptococcus*, *Ochrobacterium*, and *Pseudomonadaceae Pseudomonas* exhibited higher abundance in the EOC tissues (Figure 2). Since the intratumour microbiota of EOC has a higher alpha and beta diversity, we further isolated the specific EOC-related bacteria and obtained 81 *P. acnes* isolates. Based on our previous research that *P. acnes* occurs with the highest frequency in EOC patients, we speculate that *P. acnes* is a key strain in facilitating EOC progression. *P. acnes* is a typical opportunistic bacterium commonly occurring on the skin, especially associated with acne vulgaris [36]. It is confirmed that *P. acnes* can induce intervertebral disc degeneration by promoting iNOS/NO and COX-2/PGE2 activation via the ROS-dependent NF-κB pathway, and recent studies have also highlighted its role in oncogenesis [37]. Reports on isolation of *P. acnes* from prostate cancer indicated a link between this microorganism and etiopathogenesis of hypertrophic prostatitis, suggesting that *P. acnes* promotes cell proliferation and malignant transformation [38]. This provided clues for revealing the role of intratumoural microbiota during the development of EOC.

The Tingtao Chen’s email address has been updated to chentingtao1984@163.com.

The authors apologise for any inconvenience caused and state that the scientific conclusions are unaffected. This correction was approved by the Academic Editor. The original publication has also been updated.
