# Supplementary material for: Endogenous Propionibacterium acnes Promotes Ovarian Cancer Progression via Regulating Hedgehog Signalling Pathway
Source: Cancers (Basel). 2022 Oct 22;14(21):5178. doi: 10.3390/cancers14215178 (PMC9658903; doi:10.3390/cancers14215178)
Supplement: Supplementary file 1 [file cancers-14-05178-s001.zip › cancers-1936232-supplementary.pdf]

Table S1. Characteristics of patients with epithelial ovarian tumour.

**Characteristics**	**EBOT**	**EOC**
Total number	10	10
Gender(no.)		
Female	10	10
Male	0	0
Age (years, mean ± SD)	39.00 ± 3.70	50.80 ± 4.40
Weight (kg, mean ± SD)	55.50 ± 1.54	53.20 ± 1.52
BMI (mean ± SD)	20.52 ± 0.36	20.01 ± 0.27

Table S2. Chemicals information.

**Chemicals**		
Tryptic soy Broth (TSB)	Becton,Dickinson and Company	Cat#0350681
Man Rogosa Sharpe (MRS)	Sangon Biotech	Cat#M8330
Cause's Synthetie Agar	Solarbio	Cat#LA7000
Brain Heart Infusion (BHI)	Sangon Biotech	Cat# B530122-0010
Ampicillin	Sangon Biotech	Cat# B540124-0500
Vancomycin hydrochloride	Sangon Biotech	Cat# A100990-0100
Neomycin trisulfate salt hydrate	Sangon Biotech	Cat# A610366-0100
metronidazole	Sangon Biotech	Cat# A600633-0025

Table S3. The grouping treatment of animal.

	**Antibiotic mixture**	**Intratumoral bacteria injection**	**GANT61 injection**
Group M	−	−	−
Group M-A	+	−	−
Group M-A-BMBT	+	+	−
Group M-A-MMBT	+	+	−
Group M-A-SBT	+	+	−
Group M-A-MBT	+	+	−
Group M-A-G	+	−	+
Group M-A-SBT-G	+	+	+
Group M-A-MBT-G	+	+	+

Table S4. The information of primers.

cytokines	Forward prime Sequences	Reverse prime Sequences
GAPDH	5’-AGCCAAAAGGGTCATCATCT-3’	5’-GGGGCCATCCACAGTCTTCT-3’
TNF α	5’-GTGGAACTGGCAGAAGAGGCA -3’	5’-AGAGGGAGGCCATTTGGGAAC -3’
IL-1β	5’-GTGTCTTTCCCGTGGACCTTC -3’	5’-GCTTAGGCATAACGCACT-3’
IL-6	5’-GAAATCGTGGAAATGAG -3’	5’-GCTTAGGCATAACGCACT -3’

Table S5. The information of each specific antibody.

protein	Product Catalog	brand	Dilution ratio
β-actin	4970S	Cell Signaling Technology	1:1000
Gli1	66905-1-Ig	Proteintech	1:1000
Gli2	18989-1-AP	Proteintech	1:1000
Smo	66851-1-Ig	Proteintech	1:2000
Shh	20697-1-AP	Proteintech	1:500
Ptch1	821180	ZEN BIO	1:1000

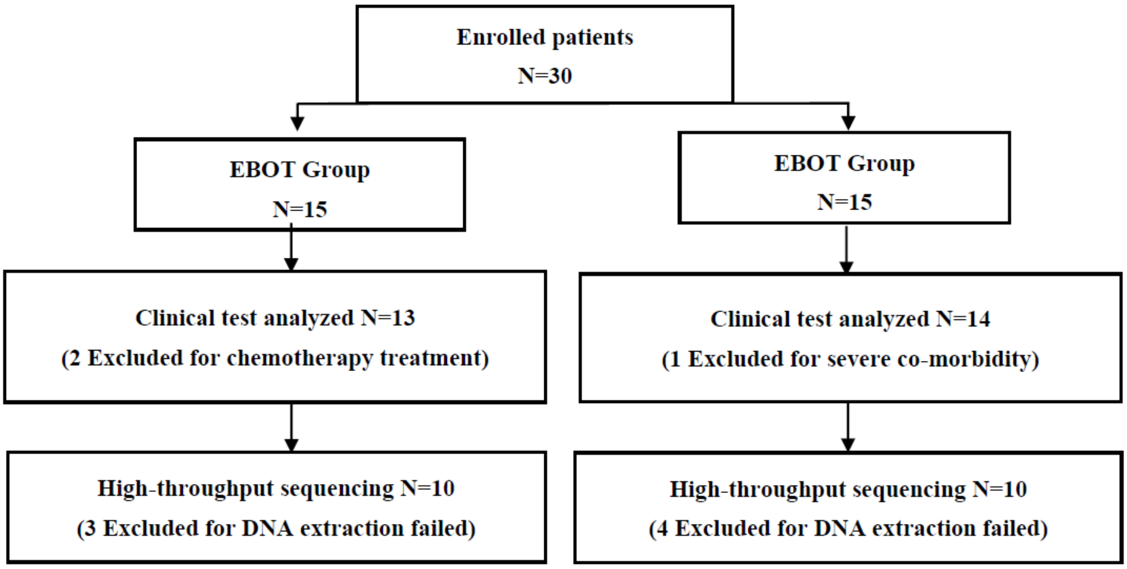

Figure S1. This study initially included 30 patients, of which 3 were excluded due to chemotherapy treatment and severe co-morbidity. Thereafter, 27 patients were collected for bacterial and microbial DNA extraction. During this process, 7 patients failed to extract DNA and were excluded.

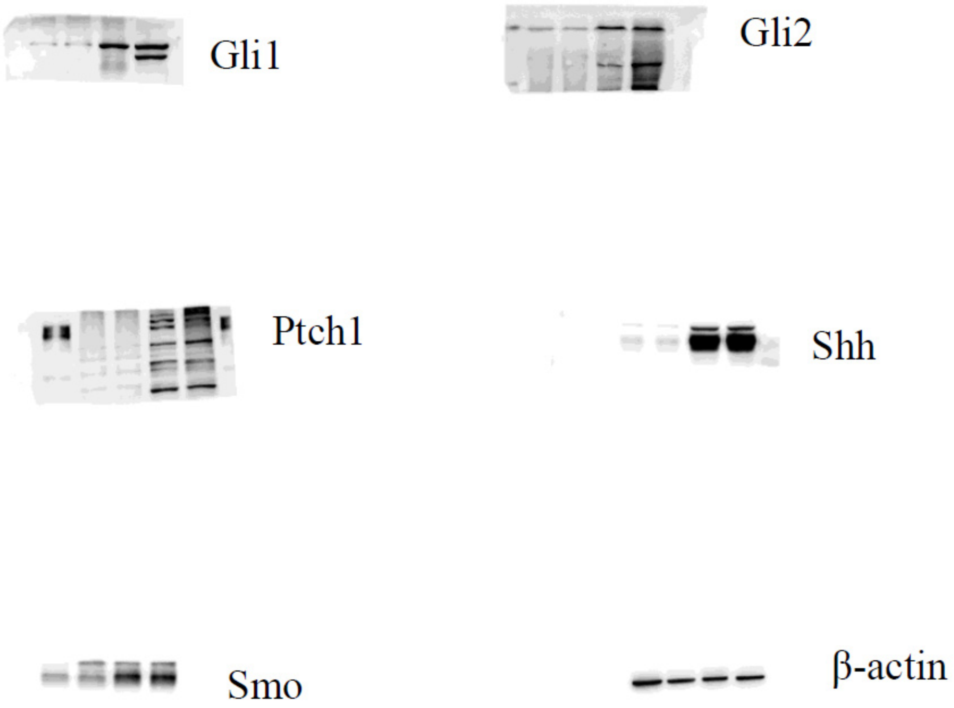

Figure S2. Original western blot images. Figure 5F.

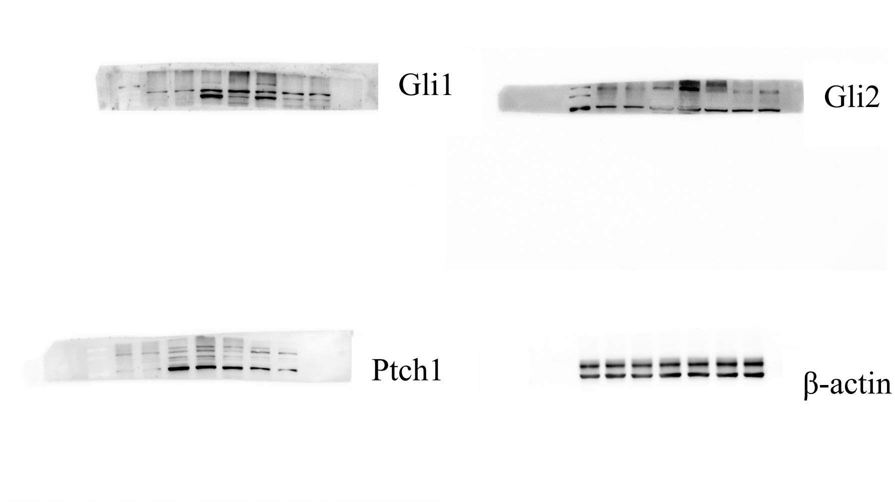

Figure S3. Original western blot images. Figure 6D.

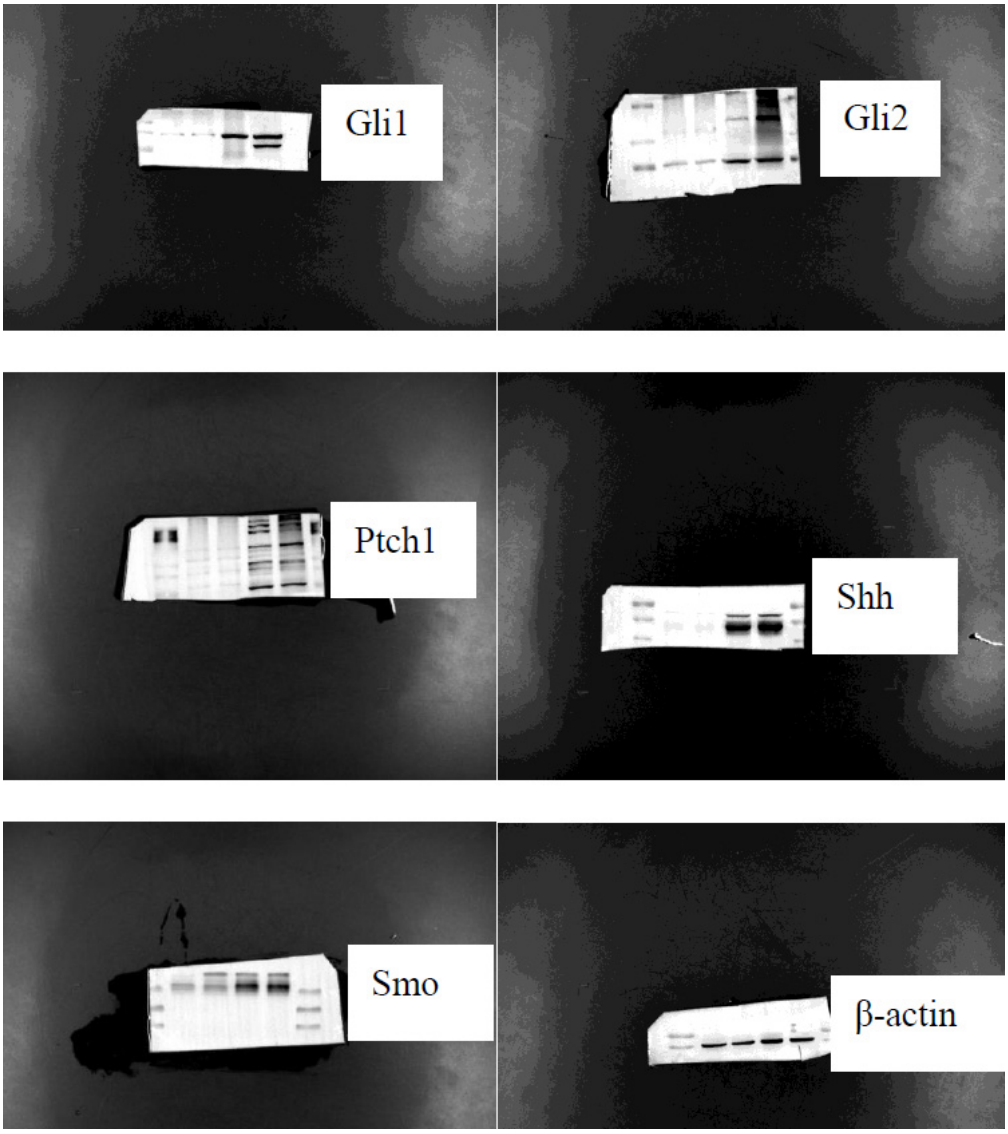

Figure S4. The uncropped blots and molecular weight markers for Figure 5F.

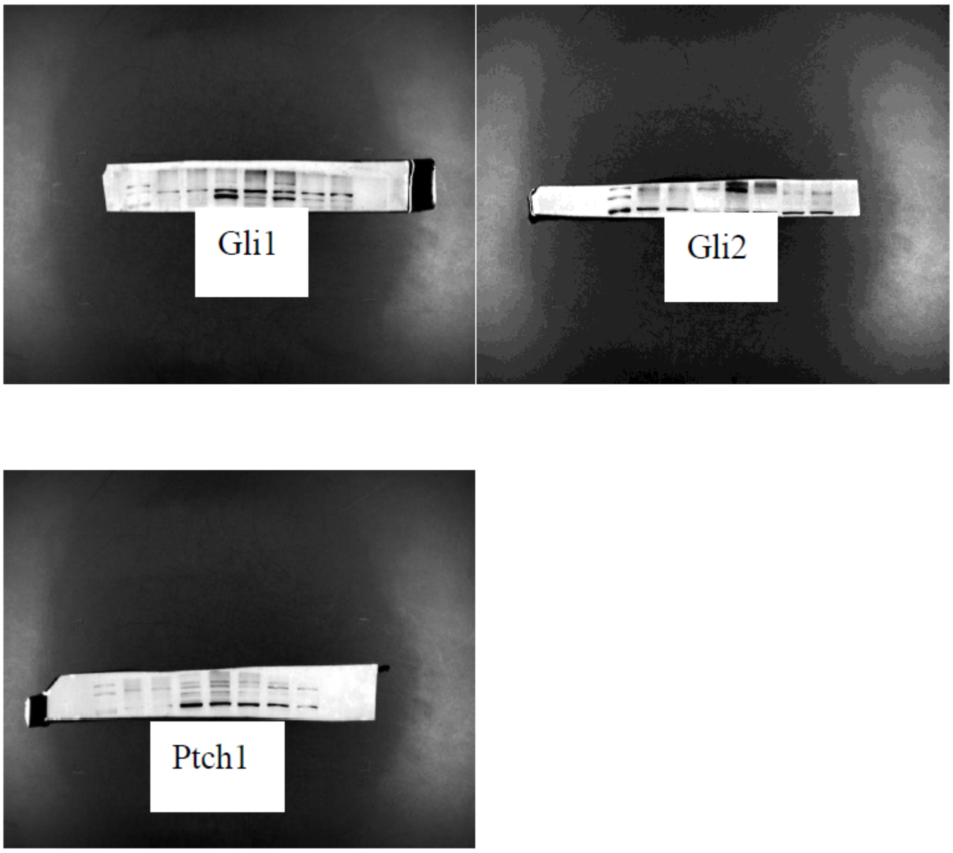

Figure S5. The uncropped blots and molecular weight markers for Figure 6D.
